# Identifying Therapeutic Targets for Bronchial Asthma: Systematic Druggable Genome‐Wide Mendelian Randomization

**DOI:** 10.1002/hsr2.71674

**Published:** 2026-01-21

**Authors:** Haijiao Wang, Peng Zhang, Hongpeng Yu, Li Shi, Tan Wang

**Affiliations:** ^1^ Changchun University of Chinese Medicine Changchun China; ^2^ The Affliated Hospital to Changchun University of Chinese Medicine Changchun China

**Keywords:** blood eQTL, bronchial asthma, bronchial asthma GWAS, druggable target genes, lung eQTL, Mendelian randomization

## Abstract

**Background:**

The treatment and prevention of bronchial asthma continue to present significant challenges. Mendelian randomization (MR) has been extensively employed to identify novel therapeutic targets. Consequently, we conducted a systematic MR analysis across the druggable genome to identify potential therapeutic targets for asthma.

**Aims:**

Exploring new potential therapeutic targets for the treatment of asthma.

**Methods:**

We obtained data on druggable genes and screened genes within lung expression quantitative trait loci (eQTL) and blood eQTL. Then, MR analysis and colocalization analysis were performed to identify genes highly associated with asthma. In addition, phenome‐wide analysis, enrichment analysis and protein–protein interaction network construction (PPI) have been carried out, providing valuable guidance for the development of more effective therapeutic targets.

**Results:**

Druggable genes were sourced from the Drug‐Gene Interaction Database (DGIdb) and supplemented by a compilation of druggable genes outlined in a review by Finan et al. The eQTL data sets are derived from eQTLGene and GTEx v8. We obtained asthma GWAS data sets from two large‐scale genotypic data sets, FinnGen, and UK Biobank. Finally, we identified six druggable genes significantly associated with asthma. Five druggable genes in whole blood (IRF1, OXER1, PSMA4, UNC13D and HLA‐DRB1) and one in lung tissue (CD226). These positive gene interactions were shown to be associated with asthma pathways by enrichment analysis. In the end, phenome‐wide analysis showed that most had no side effects.

**Conclusion:**

This study identified six potential drug targets for the treatment of asthma. This discovery provides promising insights for more effective treatments for asthma, with the potential to reduce drug development costs.

## 1 Introduction

Bronchial asthma is a hyperreactive airway disease, with recurrent episodes of wheezing, coughing, shortness of breath, and chest tightness as the main clinical manifestations, often attacked or exacerbated at night and/or in the early hours of the morning. Common triggers for their episodes include infections, exposure to allergens and/or pollutants, smoking, sudden changes in temperature, stress, and exercise, among others [1, 2]. Asthma is a serious global health problem affecting people of all ages [3]. Both disability and mortality rates place a heavy burden on global public health, with approximately 100 million people worldwide expected to be affected by 2025.

The current treatment and prevention of asthma remain extremely challenging. Although in recent years, significant progress has been made in the study of asthma genetics, revealing several potential genetic factors and therapeutic targets, for example, a Mendelian randomization study found that elevated levels of histone L2 (cathepsin L2) were significantly associated with an increased risk of asthma, which provided new insights into the pathomechanisms of asthma [4]. In addition, Janus kinase 1 (JAK1) inhibitors such as londamocitinib (AZD4604) have shown potential in targeting both T2 and non‐T2 driven inflammatory pathways, and may provide new therapeutic options for patients who do not respond well to inhaled glucocorticoids (ICS) [5]. In addition, in studies of single‐gene disorders, JAK1 gain‐of‐function (GOF) variants have been found to be associated with early‐onset severe atopic dermatitis and asthma, and JAK inhibitors may be an effective treatment for these patients [6]. These therapies offer significant convenience for asthma patients, there are still some problems, for example, the side effects and high cost of biologically targeted drugs and the fact that asthma cannot be cured, especially resistant refractory asthma, which requires high doses of inhaled glucocorticoids and systemic hormones [7]. Even so, it′s hard to control. Therefore, there is a need to continue exploring potential drug targets for the treatment of asthma.

Incorporating genetic insights into drug development may present a novel approach. Although genome‐wide association studies have been very effective in identifying single nucleotide polymorphisms (SNPs) associated with asthma risk [8]. However, in the absence of substantial downstream analysis, the method is limited in its ability to clearly and directly identify causative genes or to drive drug development [9, 10]. Approximately 30,000 genes in the human genome express drug‐binding proteins, ready‐to‐use pharmacogenomes [11], is a gene or gene product that is known or predicted to interact with therapy, encodes proteins, or regulates gene expression that can provide evidence for potential drug targets. Gene expression levels can be conceptualized as a form of lifelong exposure, reflecting the continuing influence of genetic and environmental factors on cellular and physiological processes throughout an individual's lifespan, expression quantitative trait loci (eQTL) located near druggable genes often act as proxies for these exposures. To address the limitation of genome‐wide association studies (GWAS) in directly identifying disease‐causing genes, we integrated aggregated statistics from GWAS and eQTL data sets of druggable genes to discover novel therapeutic targets. Mendelian randomization (MR) was effectively applied to the pooled data in this study [12].

In this study, we conducted a comprehensive MR analysis across the druggable genome to identify potential therapeutic targets for asthma. First, we obtained data on druggable genes and screened blood and lung tissue for eQTL, which were then analyzed by MR with asthma GWAS data to identify genes highly associated with asthma. Subsequently, we performed a colocalization analysis to ensure the robustness of the results. For important genes in blood and lung, enrichment analysis, protein–protein interaction network construction(PPI), in addition to phenome‐wide analysis to explore the relationship between shared potential therapeutic targets and other features, which offer valuable insights for the development of more effective and targeted therapeutic agents.

## 2 Materials and Methods

**Figure 1:**
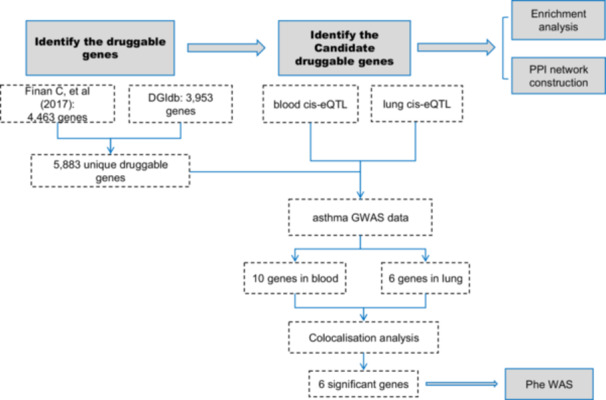
Overview of this study design.

This study used publicly available, summarized data from previous GWAS, and all original studies were ethically approved and detailed with relevant citations, so no additional approvals were required for this study. The current study was conducted in accordance with the STROBE‐MR checklist attached as supplemental material. The overview of this study is presented in Figure 1.

### 2.1 Data Source

#### 2.1.1 Druggable Genes

Drug genes are derived from the Drug‐Gene Interaction Database (DGIdb, https://www.dgidb.org/) [13] (Table S1) and comprehensive review [11]. DGIdb provides an understanding of drug–gene interactions and potential medicinal properties. We accessed DGIdb's “Category Data”, which was updated in February 2022. Furthermore, we utilized the list of druggable genes provided in a review written by Finan et al. [14] (Table S2). By integrating druggable genes from two distinct sources, a broader spectrum of druggable genes can be obtained (Table S3). These genes have been used in previous studies [15] (Table 1).

**Table 1:** Data source of exposure and outcome.

Data set	Sample size	Ethnicity	Consortium	Download site
Blood cis‐eQTL	31684	European	eQTLgen	https://eqtlgen.org/
Lung cis‐eQTL	515	European	GTEx consortium	https://gwas.mrcieu.ac.uk/
Asthma discovery	275,273 (27,012cases/248,261 controls)	European	FinnGen	https://www.finngen.fi/en
Asthma replication	463,010 (1877 cases/461,133 controls	European	UK Biobank	http://www.ukbiobank.ac.uk
DGIdb 4.0	NA	NA	Freshour et al. (2020)	https://www.dgidb.org/downloads.
Prior druggable gene	NA	NA	Finan et al. (2017)	PMID: 28356508

#### 2.1.2 eQTL Data Sets

Blood eQTL data set from eQTLGen (https://eqtlgen.org/) [16]. The study provides cis‐eQTL for 16,987 genes from 31,684 blood samples of healthy individuals of European ancestry. We obtained fully significant (false discovery rate (FDR) less than 0.05) cis‐eQTL results along with allele frequency information (Table 1). We obtained eQTL data for lung tissue from GTEx v8 (https://www.gtexportal.org/home/) (Table 1).

#### 2.1.3 Bronchial Asthma GWAS Data Sets

We obtained GWAS data on asthma from a FinnGen database (https://www.finngen.fi/en). FinnGen is a public database for the study of genomics, with a huge sample size of up to 500,000 and is constantly updated, providing a convenient resource for studying the genetics of our diseases. GWAS data for asthma in this database included 27,012 patients and 248,261 controls.

To replicate the analysis, we obtained genetic associations for asthma from the UK Biobank (UKBB) (http://www.ukbiobank.ac.uk). UKBB is a large cohort study of a whole‐fund group of linked biomedical databases containing 500,000 European subjects. The UKBB asthma GWAS data included 1877 patients and 461,133 controls (Table 1).

### 2.2 Instrumental Variables Selection

We used cis‐eQTL of druggable genes in our analysis as exposure. To ensure the robustness and reliability of our findings, the instrumental variables (IVs) used in the MR analysis had to fulfill three key assumptions. Based on these assumptions, we obtained comprehensive cis‐eQTL results (characterized by SNP‐gene distances < 1 Mb and false discovery rates [FDR] < 0.05) and allele frequency data from the eQTLGen and GTEx v8 consortia. To further mitigate the potential impact of pleiotropy and weak tools on our results, we used genome‐wide significance values (*p* < 0.001) and excluded IVs with F‐statistics < 10. Finally, to ensure that each selected significant SNP is independent and to exclude the effect of pleiotropy due to linkage disequilibrium (LD), we set the LD parameter to r2 = 0.001, kb = 10,000 and used the clump function of the TwoSampleMR package (0.6.8) to obtain IVs.

### 2.3 Statistic Analysis

#### 2.3.1 Mendelian Randomization

To ensure the integrity of our IVs, we harmonized the genetic associations with both exposures and outcomes by aligning effect alleles, subsequently excluding palindromic variants. Causal relationships were evaluated using multiple analytical models, including inverse variance weighted (IVW), MR‐Egger, simple mode, weighted median, and weighted mode approaches [17]. The *p* value of druggable genes was FDR‐corrected and is considered statistically significant at FDR < 0.05 of the causal effects. Furthermore, we employed Cochran's Q statistic to assess heterogeneity among the IVs and utilized MR‐Egger intercept to identify the presence of horizontal pleiotropy. To detect and correct potential outliers, we applied the MR‐PRESSO (Mendelian Randomization Pleiotropy RESidual Sum and Outlier) method [17].

#### 2.3.2 Colocalization Analysis

Occasionally, a SNP can reside within multiple gene regions. In instances where an SNP includes eQTL information for several genes, its effect on a disease such as asthma may be confounded by these different genes. To address this, colocalization analysis was employed to determine whether asthma and the eQTLs might share causal genetic variants. For significant MR results from the discovery phase, we conducted colocalization analysis for asthma' risk and SNPs within ±1MB of each gene's transcription start site (TSS) in eQTLs, using thresholds of P1 = 1 × 10^−4^, P2 = 1 × 10^−4^, and P12 = 1 × 10^−5^ [18]. Here, P1 represents the probability that a given SNP is associated with asthma, P2 represents the probability that a given SNP is a significant eQTL, and P12 represents the probability that a given SNP is both associated with asthma and is an eQTL. Posterior probabilities (PP) were utilized to assess the support for various hypotheses, labeled as PPH0 through PPH4: PPH0 indicates no association with any trait; PPH1 indicates association with gene expression but not asthma risk; PPH2 indicates association with asthma risk but not gene expression; PPH3 indicates association with both asthma risk and gene expression, with distinct causal variation and PPH4 indicates association with both asthma risk and gene expression, sharing a common causal variant. Due to the limitations of colocalization analysis, subsequent analyses were confined to genes where the ratio PPH4/(PPH3 + PPH4) was 0.75 or greater [19].

#### 2.3.3 Enrichment Analysis

To examine the functional characteristics and biological significance of the identified prospective druggable genes, we employed the R package “clusterProfiler” (version 4.10.1) [20] to conduct Gene Ontology (GO) and Kyoto Encyclopedia of Genes and Genomes (KEGG) enrichment analyses. The GO analysis encompasses three domains: Biological Process (BP), Molecular Function (MF), and Cellular Component (CC). KEGG pathway analysis provides insights into metabolic pathways.

#### 2.3.4 Protein–Protein Interaction Network Construction

Protein–protein interaction (PPI) networks facilitate the visualization of relationships among proteins encoded by significant druggable genes. We constructed these PPI networks using the STRING database (https://string-db.org/), establishing a minimum confidence score threshold of 0.4 for interaction, while retaining all other parameters at their default settings [21].

#### 2.3.5 Phenome‐Wide Association Analysis

To investigate potential side effects linked to six identified druggable genes, we utilized summary statistics from disease outcomes in both the UKBB cohort and the FinnGen R11 cohort to conduct phenome‐wide Mendelian randomization (phe‐MR) analyses. In light of statistical power requirements, we selected 2514 traits from the UKBB, each comprising over 1000 cases, and 2440 traits from the FinnGen cohort for our phe‐MR evaluations. A false discovery rate (FDR) of a value of less than 0.05 was employed to indicate statistical significance.

## 3 Results

### 3.1 Druggable Genome

We retrieved 3953 druggable genes from the DGIdb (Table S1). In addition, we obtained 4463 druggable genes from a previous review (Table S2) [14]. Following the integration of the data, we obtained 5883 unique druggable genes named by the Gene Nomenclature Committee of the Human Genome Organization for subsequent analysis (Table S3).

### 3.2 Screening the Causal Genes for Bronchial Asthma and Identifying Candidate Blood‐Specific Druggable Genes

**Figure 2:**
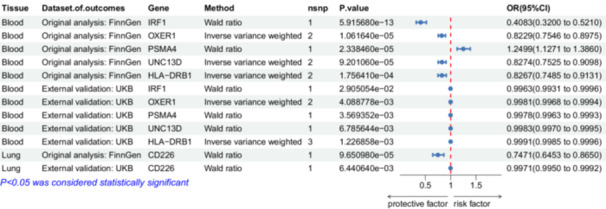
Forest plot of six genes associated with commonly used medications for asthma from blood and lung.

To make the results more accurate and convincing, we selected genes with FinnGen discovery cohort FDR < 0.05 and original *p*< 0.05 of the UKBB validation cohort as MR positive results, and screened out 10 druggable genes (IRF1, SMAD3, OXER1, TCF19, PSMA4, AXIN2, UNC13D, HLA‐DRB1, PNMT, AGER). IRF1 (Finn, odds ratio (OR) = 0.408, 95% confidence interval (CI) = 0.320–0.521, *p*< 0.001; UKBB, OR = 0.996, 95%CI = 0.993–1.000; *p*= 0.39), SMAD3 (Finn, OR = 0.728, 95%CI = 0.638–0.830, *p*= 0.006; UKBB, OR = 0.998, 95%CI = 0.996–1.000; *p*= 0.41), OXER1 (Finn, OR = 0.823, 95%CI = 0.755–0.897, *p*= 0.007; UKBB, OR = 0.998, 95%CI = 0.997–0.999; *p*= 0.20), TCF19 (Finn, OR = 0.928, 95%CI = 0.898‐0.959, *p*= 0.02; UKBB, OR = 1.005, 95%CI = 1.002–1.007; *p*= 0.02), PSMA4 (Finn, OR = 1.250, 95%CI = 1.127–1.386, *p*= 0.02; UKBB, OR = 0.998, 95%CI = 0.996–0.999; *p*= 0.18), AXIN2 (Finn, OR = 1.143, 95%CI = 1.072–1.219, *p*= 0.03; UKBB, OR = 1.001, 95%CI = 1.000–1.002; *p*= 0.22), UNC13D (Finn, OR = 0.827, 95%CI = 0.752–0.910, *p*= 0.06; UKBB, OR = 0.998, 95%CI = 0.997–1.000; *p*= 0.34), HLA‐DRB1 (Finn, OR = 0.827, 95%CI = 0.749–0.913, *p*= 0.33; UKBB, OR = 0.999, 95%CI = 0.998–1.000; *p*= 0.91), PNMT (Finn, OR = 1.725, 95%CI = 1.296–2.29, *p*= 0.03; UKBB, OR = 1.004, 95%CI = 1.000–1.008; *p*= 0.52), AGER (Finn, OR = 1.237, 95%CI = 1.105–1.385, *p*= 0.04; UKBB, OR = 1.006, 95%CI = 1.002–1.010; *p*= 0.07) (Tables S4–S6, Figure 2).

**Figure 3:**
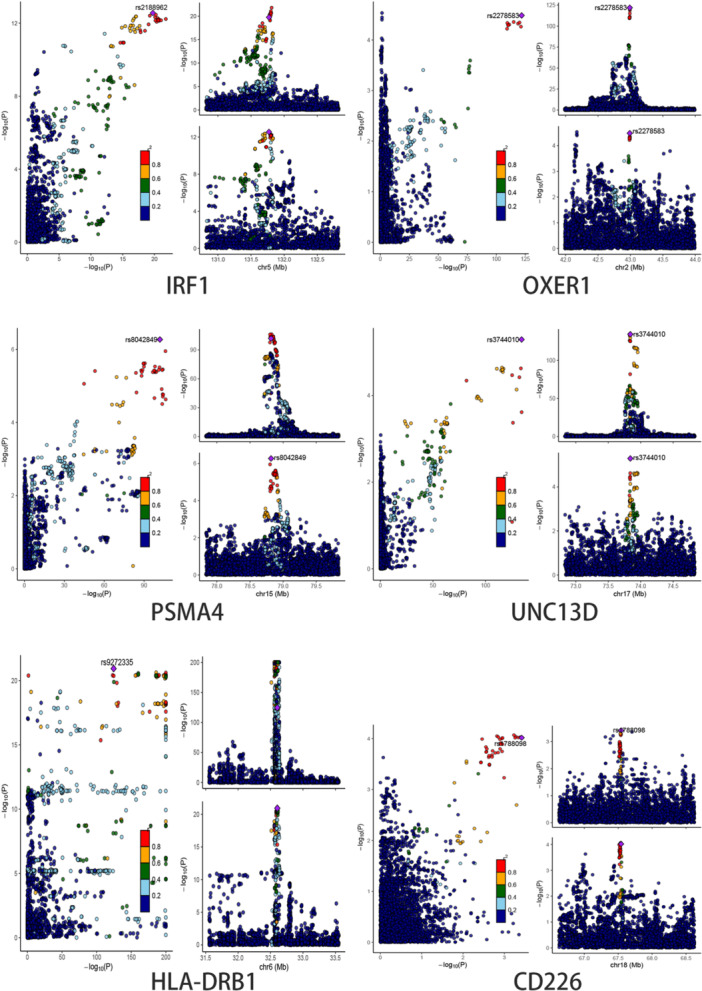
Colocalization results of five significant genes from blood and one significant gene from lung.

To further determine the likelihood of SNPs sharing causal genetic variation associated with blood eQTL and asthma, we performed Bayesian colocalization analyses using blood cis‐eQTL data for the 10 potentially druggable genes described above and identified five drug targets. IRF1 (PPH4/PPH3 + PPH4 = 0.975), OXER1(PPH4/PPH3 + PPH4 = 0.967), PSMA4(PPH4/PPH3 + PPH4 = 0.972), UNC13D (PPH4/PPH3 + PPH4 = 0.989) and HLA‐DRB1(PPH4/PPH3 + PPH4 = 0.948) expression had significant colocalization associations with asthma. Therefore, five potentially druggable genes were identified using MR and Bayesian colocalization analyses that showed evidence of common genetic effects between blood eQTL and asthma risk (Table S10, Figure 3).

### 3.3 Candidate Lung‐Specific Druggable Genes for Bronchial Asthma

Similarly, in lung tissue, we chose the above two databases, Finn found genes with cohort FDR < 0.05 and UKBB validation cohort raw *p* < 0.05 as MR positive results, and screened a total of six druggable genes (MICA, MSH5, RAD50, MICB, CD226, PNMT). MICA (Finn, OR = 1.094, 95%CI = 1.060–1.130, *p* < 0.001; UKBB, OR = 1.001, 95%CI = 1.000–1.001; *p* = 0.25), MSH5 (Finn, OR = 0.660, 95%CI = 0.561–0775, *p* = 0.004; UKBB, OR = 0.995, 95%CI = 0.993–0.998; *p* = 0.05), RAD50 (Finn, OR = 1.244, 95%CI = 1.128–1.371, *p* = 0.008; UKBB, OR = 1.002, 95%CI = 1.001–1.004; *p* = 0.07), MICB (Finn, OR = 1.101, 95%CI = 1.053–1.150, *P* = *0.03*; UKBB, OR = 1.001, 95%CI = 1.001–1.002; *p* = 0.05), CD226 (Finn, OR = 0.747, 95%CI = 0.645–0.865, *p* = 0.07; UKBB, OR = 0.997, 95%CI = 0.995–0.999; *p* = 0.32), PNMT (Finn, OR = 1.263, 95%CI = 1.117–1.427, *p* = 0.03; UKBB, OR = 1.002, 95%CI = 1.000–1.004; *p *= 0.03) (Tables S7–S9, Figure 2).

To further determine the likelihood of shared causal genetic variation in SNPs associated with eQTLs and asthma in lung tissue, we performed Bayesian colocalization analyses using cis‐eQTL data from lung tissue for the six potentially druggable genes described above and identified one drug target. Expression of CD226 (PPH4/PPH3 + PPH4 = 0.751) showed a significant colocalization association with asthma. Thus, one potentially druggable gene was identified using MR and Bayesian colocalization analyses, demonstrating a common genetic effect between eQTL and asthma risk in lung tissue (Table S10, Figure 3).

### 3.4 Enrichment Analysis

**Figure 4:**
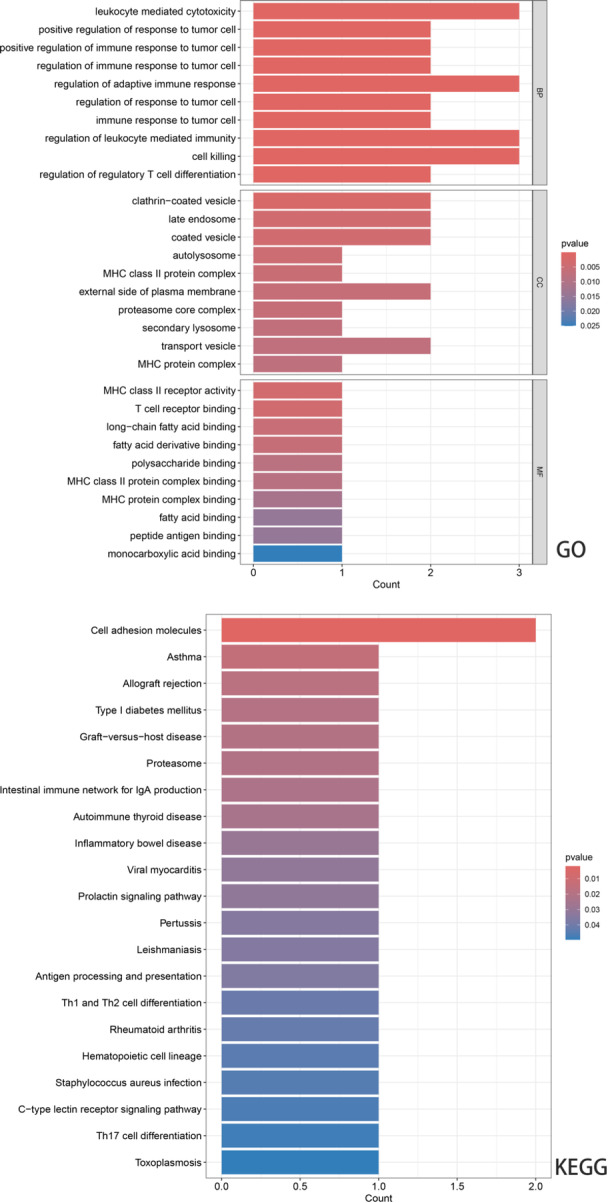
GO and KEGG enrichment results.

**Figure 5:**
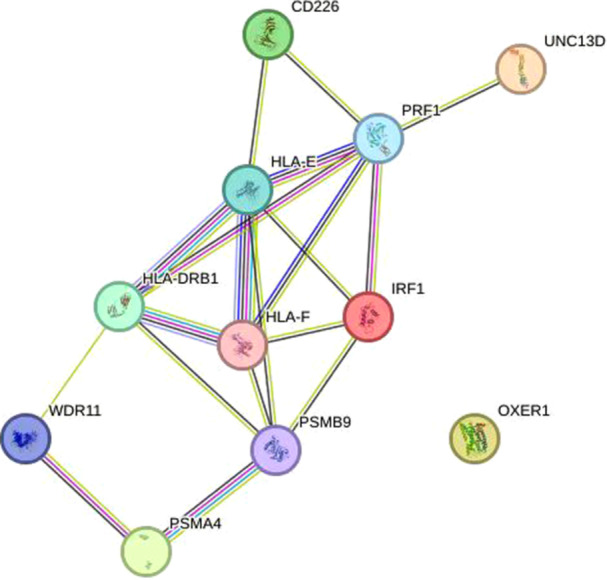
PPI network built with STRING.

We performed functional enrichment analysis and PPI network construction for six genes significantly associated with asthma. The GO enrichment analysis results indicated that the important biological process (BP) terms included leukocyte mediated cytotoxicity, regulation of leukocyte‐mediated immunity, etc.; the cellular component (CC) terms were mainly associated with clathrin ‐coated vesicle, external side of plasma membrane and so on; Molecular function (MF) terms included MHC class II receptor activity, T cell receptor binding, and others. In addition, KEGG pathway analysis showed that these interacting genes were mainly concentrated in asthma and cell adhesion molecular pathways (Figure 4). The STRING database is utilized to construct the PPI network, where the interaction pathways are shown in Figure 5.

### 3.5 Phe‐MR Analysis of Drug Candidate Genes for Bronchial Asthma‐Related Phenotypes

Phe‐MR analysis of drug candidate genes for asthma‐related phenotypes to elucidate the associations between the main evidence genes and specific phenotypes, we performed phe‐MR analyses to find associations between different genes and different phenotypes that are likely to be potential therapeutic targets as a side effect‐indicating that there may be a side effect of these druggable targets. No significant side effect was found in all ph‐mr results. In our analysis, we employed an FDR threshold of less than 0.05 to determine statistical significance. This threshold was applied consistently across the phenome‐wide Mendelian randomization (phe‐MR) analyses to identify any potential associations between the identified druggable genes and a broad range of phenotypes. Specifically, we evaluated 2514 traits from the UK Biobank cohort and 2440 traits from the FinnGen cohort, ensuring a comprehensive assessment of possible side effects.

## 4 Discussion

The study combined existing druggable gene targets with asthma GWAS data using MR and colocalization analysis. Five druggable genes significantly associated with asthma were identified in whole blood (IRF1, OXER1, PSMA4, UNC13D, and HLA‐DRB1) and one in lung tissue (CD226). We enriched and analyzed these six significant genes and constructed PPI networks to elucidate the biological significance and interaction mechanisms underlying these drug targets. Finally, phe‐MR analysis was performed to further validate the potential side‐effects of these important druggable genes.

IRF1 is a nuclear transcription factor that can be involved in the expression of a variety of genes via relevant signaling channels and is involved in cell differentiation, cellular immunity, and other physiopathological processes. Tliba et al. suggested that high expression of IRF1 results in decreased responsiveness to glucocorticoids used to treat asthma [22]. This mechanism of action is associated with GLCCI 1 deficiency, which upregulates IRF 1 and IRF 3 in proportion to it, thereby attenuating dexamethasone sensitization of mouse asthma epithelial cells to glucocorticoid resistance [23]. A genome‐wide study, the IRF1 locus is a strong candidate region for specific asthma susceptibility [24]. It can regulate downstream inflammation and cell death and serve as a target gene to address inflammatory and infectious diseases linked to dysregulated inflammatory cell death [25]. Many previous studies have shown that the IRF1 gene has an inflammatory effect, leading to acute asthma attacks [26]. Therefore, it can be used to assist in the treatment of acute exacerbations of asthma by controlling IRF1 levels [27].

OXER1 is an inflammatory receptor that plays a key role in multiple signaling pathways. It can induce hypoxia and transmit protein information through downstream markers [28]. OXE receptor, encoded by the OXER1 gene, may be a target for the treatment of asthma, for which an animal study was carried out in the selection of an OXE receptor antagonist. It inhibits allergen‐induced inflammation in the lungs, and finally, the study suggests that OXE receptor antagonist may be a useful therapeutic agent for the treatment of asthma [29]. Asthma is mediated by the involvement of eosinophils, neutrophils, which are associated with allergy and inflammation. OXER1 was previously reported to be highly expressed in leukocytes, macrophages, monocytes and cancer cells, which and it has been shown that 5‐LOX is directly involved in lung cancer progression. The OXER1 receptor is a major marker downstream of the 5‐LOX pathway. The use of various blockers of 5‐LOX and OXER1 has led to improved efficacy in the treatment of various cancers. Agents that inhibit the LOX‐mediated signaling pathway have since been used to treat inflammatory diseases such as asthma and arthritis [30]. Further evidence that the OXER1 gene is associated with the development of asthma may provide a new target for asthma treatment.

PSMA 4 has an important role in inflammatory regulation and response to stress, which may be an important target for the treatment of severe infections. An MR study analyzed that PSMA 4 has been identified as a new therapeutic target for COPD [31], PSMA 4 upregulation leads to the progression of lung function in COPD, which exacerbates the disease, and since asthma has an overlapping component with chronic obstructive pulmonary disease, downregulation of PSMA 4 expression by targeting PSMA 4 can help to control asthma and airway hyperresponsiveness. So PSMA 4 may be a target for the treatment of asthma.

The UNC13 family proteins are evolutionarily conserved, with recent studies highlighting multiple roles for UNC13D in mediating the secretion of cytotoxic granules by immune cells [32, 33, 34, 35]. It is also involved in genetic disease pathology. Patients with familial phagocytic lymphohistiocytosis type 3 (FHL3) with low UNC13D levels show a high inflammatory response [36]. Mutations in the UNC13D gene rarely involve the lungs, but a previous rare case reported lymphocytic interstitial pneumonitis in an adult FHL3 patient with improvement of the clinical symptoms after the application of corticosteroids and immunosuppressant treatment [37], which shows that UNC13D, as a target gene used to treat lung diseases, needs to be further clarified.

HLA‐DRB1 belongs to the HLA class IIβ‐chain parallel genes, class II molecules are expressed in antigen‐presenting cells. There are hundreds of DRB1 alleles, and the relationship between different alleles and the disease varies. Li et al. identified the HLA‐DR gene region as the region of the asthma‐occurring gene mutation through a genome‐wide association study of asthma [38]. The HLA‐DRB 1 allele has been actually associated with asthma [39, 40, 41]. The DRB1*15 is negatively associated with the risk of asthma [42]. Allergens are important factors leading to acute asthma attacks. Donfack et al. studied the HLA regional alleles and found that DRB1*0101, DRB 1*0102 alleles pose a risk of cockroach sensitization, thus elucidating that elucidation of HLA‐DRB 1 can be used for the treatment of atopic asthma at the molecular level [43].

The IRF1‐HLA‐DRB1 axis represents a key immunoregulatory pathway in asthma pathogenesis. IRF1 transcriptionally regulates MHC class II expression, including HLA‐DRB1, through activation of the CIITA promoter. This interaction enhances antigen presentation in airway epithelium, potentially amplifying Th1/Th17 responses in severe asthma. Concurrently, IRF1 deficiency exacerbates neutrophilic inflammation in pulmonary infection models [44], suggesting its protective role in maintaining immune homeostasis. Therapeutic modulation of this axis may therefore correct aberrant adaptive immunity while suppressing pathogenic neutrophilia.” We identified the OXER1‐UNC13D axis as a key hub in the amplification of asthma inflammation. oXER1 activates UNC13D‐dependent degranulation via calcium signaling, while UNC13D deficiency attenuates airway hyperresponsiveness [45]. Targeting this pathway (e.g., the OXER1 antagonist S‐005) may be particularly indicated in patients with severe asthma with elevated levels of 5‐oxo‐ETE. Its interaction with the IL‐4R/TSLP pathway needs to be explored in the future to optimize the combination treatment strategy.

CD226 is a stimulatory receptor that can act on a variety of cells, including T cells, and is involved in the inflammatory response process. An experimental study found that CD226 deficiency in CD4 + T cells attenuated airway hyperresponsiveness in asthma in mice, thus validating that targeting CD226 may provide new ideas for the clinical treatment of asthma [46]. In 2023, a study on the mechanism of asthma similarly demonstrated that down‐regulation of CD226 reduces allergic responses in the asthmatic airways, providing evidence for novel therapeutic approaches to asthma [47]. A number of subsequent studies have also confirmed that CD226 can be a therapeutic target for asthma, providing a basis for subsequent studies.

This study possesses several strengths, notably its comprehensive collection of eQTL data, a large number of asthma cases, reciprocal validation of two independent outcome data sets, and supportive colocalization analyses. Enrichment analysis and PPI elucidated the functional characterization and regulatory relationships of these target genes, suggesting asthma drug targets supported by robust evidence for potential avenues for asthma drug development. Certain limitations should be taken into account when interpreting our findings. First, our analysis was limited to individuals of European descent and was not generalizable. Second, the study focused primarily on cis‐eQTL and their association with asthma, potentially overlooking other factors contributing to the complexity of the disease. Third, although enrichment analysis provides valuable insights, it is accompanied by inherent limitations because it relies on predefined genomes or pathways that may not include all possible biological mechanisms or interactions. Finally, the need for further clinical validation of our findings and the lack of data from clinical relevance studies is a significant limitation.

## 5 Conclusion

Our study aimed to discover new targets for the treatment of asthma, and this study identified six druggable genes (IRF1, OXER1, PSMA4, UNC13D, HLA‐DRB1, and CD226) that provide genetic evidence for the development of drugs for asthma; however, additional studies are needed to assess the feasibility of these six identified druggable genes as therapeutic drugs for asthma. OXER1's role in leukotriene signaling suggests potential utility of existing leukotriene modifiers in genetically defined subgroups. For targets of existing drug therapies, such as ADRB2:β2‐agonists; IL4Rα/IL5 R/TSLP: monoclonal antibodies such as Dupilumab, Benralizumab, and Tezepelumab. We will optimize existing drugs (e.g., inhaled ADRB2 agonists with improved kinetics) or explore new indications. For targets lacking approved drugs (e.g., IRF1, HLA‐DRB1), we will propose specific approaches: IRF1: Development of small molecule inhibitors or gene silencing strategies (siRNA/ASO) to modulate the interferon response. Precedents exist in oncology [48]. HLA‐DRB1: Explore peptide therapies or immunomodulators targeting specific HLA‐DRB1 alleles associated with asthma risk [49]. The genetic signatures identified may aid in developing companion diagnostics. Our research highlights the necessity of integrating genetic evidence into future treatment algorithms. Doctors can utilize this framework to evaluate emerging therapies within the context of individual genetic characteristics, moving towards personalized management approaches. Although our MR analysis provides reliable genetic support for these targets, clinical validation through randomized trials is still necessary before the implementation of the treatment. We now explicitly recommend that doctors monitor ongoing research on these pathways to guide future practice.

## Author Contributions

Tan Wang and Li Shi contributed to the study conception and design. Haijiao Wang, Peng Zhang, and Hongpeng Yu performed the statistical analysis. Haijiao Wang drafted the manuscript. All authors commented on previous versions of the manuscript. All authors contributed to the article and approved the submitted version.

## Ethics Statement

The data is publicly available, ethical approval was not required, and all data have been anonymized.

## Conflicts of Interest

The authors declare no conflicts of interest.

## Transparency Statement

The corresponding author, Tan Wang, affirms that this manuscript is an honest, accurate, and transparent account of the study being reported; that no important aspects of the study have been omitted; and that any discrepancies from the study as planned (and, if relevant, registered) have been explained.

## Supporting information


**Table S1:** The potential druggable genes in DGIdb. **Table S2:** The druggable genes from Finan. C et al. **Table S3:** The final list of druggable gene symbols used in this study. **Table S4:** Mendelian randomization analysis results showed genetically predicted blood gene expression is significantly associated with ashtma in the original analysis and external validation. **Table S5:** Mendelian randomization analysis results showed associations between genetically predicted blood gene expression and ashtma in the original analysis. **Table S6:** Mendelian randomization analysis results showed associations between genetically predicted blood gene expression and ashtma in the external validation. **Table S7:** Mendelian randomization analysis results showed genetically predicted tissue‐specific gene expression is significantly associated with ashtma in the original analysis and external validation. **Table S8:** Mendelian randomization analysis results showed associations between genetically predicted tissue‐specific gene expression and ashtma in the original analysis. **Table S9:** Mendelian randomization analysis results showed associations between genetically predicted tissue‐specific gene expression and ashtma in the external validation. **Table S10:** The results of colocalization analysis.

## Data Availability

All data sources are shown in Table 1.
